# Ambient Air Pollution and Birth Weight in Full-Term Infants in Atlanta, 1994–2004

**DOI:** 10.1289/ehp.1002785

**Published:** 2010-12-14

**Authors:** Lyndsey A. Darrow, Mitchel Klein, Matthew J. Strickland, James A. Mulholland, Paige E. Tolbert

**Affiliations:** 1 Department of Environmental Health, Emory University, Atlanta, Georgia, USA; 2 Department of Civil and Environmental Engineering, Georgia Institute of Technology, Atlanta, Georgia, USA

**Keywords:** air pollution, air pollution epidemiology, birth weight, particulate matter, pregnancy outcomes

## Abstract

**Background:**

An emerging body of evidence suggests that ambient levels of air pollution during pregnancy are associated with fetal growth.

**Objectives:**

We examined relationships between birth weight and temporal variation in ambient levels of carbon monoxide, nitrogen dioxide (NO_2_), sulfur dioxide (SO_2_), ozone, particulate matter ≤ 10 μm in diameter (PM_10_), ≤ 2.5 μm (PM_2.5_), 2.5 to 10 μm (PM_2.5–10_), and PM_2.5_ chemical component measurements for 406,627 full-term births occurring between 1994 and 2004 in five central counties of metropolitan Atlanta.

**Methods:**

We assessed relationships between birth weight and pollutant concentrations during each infant’s first month of gestation and third trimester, as well as in each month of pregnancy using distributed lag models. We also conducted capture-area analyses limited to mothers residing within 4 miles (6.4 km) of each air quality monitoring station.

**Results:**

In the five-county analysis, ambient levels of NO_2_, SO_2_, PM_2.5_ elemental carbon, and PM_2.5_ water-soluble metals during the third trimester were significantly associated with small reductions in birth weight (−4 to −16 g per interquartile range increase in pollutant concentrations). Third-trimester estimates were generally higher in Hispanic and non-Hispanic black infants relative to non-Hispanic white infants. Distributed lag models were also suggestive of associations between air pollutant concentrations in late pregnancy and reduced birth weight. The capture-area analyses provided little support for the associations observed in the five-county analysis.

**Conclusions:**

Results provide some support for an effect of ambient air pollution in late pregnancy on birth weight in full-term infants.

Birth weight has long been recognized as a strong predictor of infant morbidity and mortality. Over the last 10 years, epidemiologic studies have reported relationships between ambient air pollution and measures of reduced fetal growth (Glinianaia et al. 2004; Šrám et al. 2005). To separate effects on fetal growth from effects on length of gestation, investigators have examined low birth weight (< 2,500 g) in full-term infants, birth weight adjusted for gestational age, and small for gestational age (commonly defined as birth weight below the 10th percentile for gestational age). Many studies suggest an association between fetal growth and ambient particulate matter (PM), although the gestational window of effect has not been consistent across studies. Associations with PM have been reported most commonly for early pregnancy exposures (e.g., first month, first trimester) (Gouveia et al. 2004; Liu et al. 2003; Rich et al. 2009) and late pregnancy exposures (e.g., third trimester) (Bell et al. 2007; Rich et al. 2009; Wilhelm and Ritz 2005), although other investigators have observed associations only in midpregnancy or not at all (Hansen et al. 2007; Lee et al. 2003; Mannes et al. 2005). Gaseous air pollutants have also been investigated in relation to measures of fetal growth and show similar inconsistencies. Literature reviews and meeting reports conclude that further research is warranted to clarify the gestational windows of susceptibility and to identify specific pollutants or pollutant constituents associated with fetal growth (Slama et al. 2008; Woodruff et al. 2009). There is increasing interest in traffic-related air pollutants such as carbon monoxide (CO), nitrogen oxides (NO_x_), and primary exhaust particles (e.g., elemental carbon) because of reports implicating proximity to traffic sources in reduced fetal growth (Rocha et al. 2008; Wilhelm and Ritz 2003).

Air pollution could act through several biological mechanisms to inhibit fetal growth. Pulmonary and placental inflammation, increased blood coagulation and viscosity, and altered endothelial and vascular function could compromise uteroplacental blood flow and inhibit the placental transfer of oxygen and nutrients from mother to fetus. Some of these mechanisms are thought to be involved in the well-documented relationship between maternal active and passive smoking and fetal growth restriction (Gabbe 2002; U.S. Department of Health and Human Services 2001, 2006). Studies of maternal smoking suggest that the third trimester is a particularly vulnerable exposure window (Lieberman et al. 1994; U.S. Department of Health and Human Services 2001). Furthermore, the third trimester corresponds to the period of most rapid fetal growth and fat accumulation (Gabbe 2002). Early pregnancy may also be a period of vulnerability, with abnormal reaction between trophoblast and uterine tissue around the time of implantation leading to chronic placental insufficiency throughout pregnancy (Torry et al. 2004). Evidence supporting early pregnancy effects comes from a toxicologic study that found early pregnancy exposures among mice to be the most harmful (Rocha et al. 2008).

We investigated relationships between ambient levels of air pollution during pregnancy and birth weight in a cohort of 406,627 full-term births occurring between 1994 and 2004 in the five central counties of metropolitan Atlanta, Georgia. Pollutants examined included CO, nitrogen dioxide (NO_2_), sulfur dioxide (SO_2_), ozone (O_3_), particulate matter ≤ 10 μm in diameter (PM_10_), PM_2.5_ (< 2.5 μm), and PM_2.5_ chemical component measurements that are rarely available on a daily basis and have not been previously assessed in relation to measures of fetal growth. We focused on two gestational windows of *a priori* interest based on previous air pollution studies, studies of maternal active and passive smoking, and toxicologic evidence: the first month of gestation and the third trimester. In a novel use of distributed lag models, we used a data-based approach to explore associations in each of the 9 months of gestation to identify other potentially vulnerable gestational windows.

## Materials and Methods

### Study population

We obtained vital record data for births to mothers residing in the five central counties of the Atlanta metropolitan area (Clayton, Cobb, DeKalb, Fulton, and Gwinnett counties) from the Office of Health Information and Policy, Georgia Division of Public Health. The study area included an area with a radius of 16 miles (25.7 km) at its narrowest and 32 miles (51.5 km) at its widest [see Supplemental Material, Figure 1 (doi:10.1289/ehp.1002785)]. We restricted analysis to full-term infants to separate the pathologies of reduced fetal growth from reduced pregnancy duration (i.e., preterm birth); results of our preterm birth analyses are reported separately (Darrow et al. 2009a). The cohort included singleton non-Hispanic African-American, non-Hispanic Caucasian, and Hispanic infants who reached at least 37 weeks of gestation and were born between 1 January 1994 and 31 December 2004 without major structural congenital birth defects (*n* = 417,280). Of these, 10,653 infants (2.5%) were excluded because of missing data. Gestational age was determined using the reported last menstrual period (LMP) date; if LMP date was missing, we substituted the clinical estimate (1.3% of records). After exclusions, 406,627 full-term births were eligible for analysis. Study protocols were approved by Emory University Institutional Review Board.

### Ambient air quality data

We obtained ambient air pollution levels from three sources: *a*) the U.S. EPA Air Quality System (U.S. EPA 2006), *b*) the Aerosol Research and Inhalation Epidemiology Study (ARIES) monitor located in downtown Atlanta (Hansen et al. 2006), and *c*) the Assessment of Spatial Aerosol Composition in Atlanta network (Butler et al. 2003). The daily air metrics included 1-hr maximum CO, NO_2_, and SO_2_; 8-hr maximum O_3_; and 24-hr average PM_10_, PM_2.5_, PM_2.5–10_ (PM with aerodynamic diameter from 2.5 to 10 μm), PM_2.5_ sulfate, PM_2.5_ nitrate, PM_2.5_ organic carbon, PM_2.5_ elemental carbon, and a PM_2.5_ water-soluble metals index (sum of chromium, copper, iron, manganese, nickel, and vanadium). Gaseous pollutants were available for the entire study period. Daily PM_10_ monitoring began in January 1996 and daily PM_2.5_, PM_2.5–10_, and PM_2.5_ component monitoring began in August 1998.

For CO, NO_2_, SO_2_, O_3_, PM_10_, and PM_2.5_, we calculated a population-weighted spatial average for each day in the study area using a method described in detail by Ivy et al. (2008). This approach incorporated all the monitoring data available for each pollutant on a given day in a spatial model that provided ambient pollutant levels at population census tracts, from which citywide population-weighted values were calculated robust to missing data at individual monitoring sites (Ivy et al. 2008). We used 5 CO monitors, 6 NO_2_ monitors, 5 SO_2_ monitors, 5 O_3_ monitors, 9 PM_10_ monitors, and 11 PM_2.5_ monitors to calculate the daily spatial averages. For the coarse PM measurements (PM_2.5–10_) and the PM_2.5_ component measurements, daily measurements from the centrally located ARIES monitor were used. We imputed O_3_ measurements for winters between 1994 and 1996 using a statistical model of temperature and week of year. In Bland–Altman plots, O_3_ values calculated using this prediction model showed strong agreement with the population-weighted spatial average O_3_ values in winters after 1996 when O_3_ was measured (mean difference, 0 ppb; limits of agreement, −6 to 6 ppb).

### Exposure assignment

Daily population-weighted pollutant values for five-county Atlanta were averaged over the time period corresponding to the gestational window of interest for each infant in the cohort. Using conventional obstetric notation, gestational age was calculated from the LMP date, and conception was estimated to occur on gestational day 14. For the first-month-of-pregnancy exposure window, pollutant concentrations were averaged over the 28 days beginning on the estimated conception date (gestational days 14–41). For the third-trimester exposure window, pollutant concentrations were averaged over the period beginning on gestational day 196 (28 gestational weeks) and ending on the day of birth for each infant (mean length, 83 days; range, 64–119 days). In a sensitivity analysis, we assessed pollutant effects separately for infants born at each gestational week (37–44) so that infants were grouped by the length of their third trimester and results could be compared between longer and shorter gestations. For all pollutants and gestational windows presented, an exposure was assigned only when at least 85% of days in the averaging window had available pollution data; otherwise, the exposure was set to missing.

To identify other potentially vulnerable gestational windows, we used constrained distributed lag models to assess associations for pollutant concentrations in each of the 9 months of pregnancy while controlling for pollutant concentrations in the other months of pregnancy. Although not previously applied in the context of air pollution and pregnancy outcomes (to our knowledge), distributed lag models have been used commonly in time-series air pollution studies to simultaneously assess the effects of pollution on multiple days (i.e., lags) before the health event (Schwartz 2000). Constraining the 9 month–specific effect estimates to follow the shape of a polynomial reduces the statistical noise due to the collinearity between exposures on neighboring months of pregnancy. By imposing an underlying cubic structure, we assumed that the effects of air pollution across the 9 months of pregnancy follow a smooth cubic shape (as in our other analyses, the pollutant effect in any given month of pregnancy is assumed to be linear). For this analysis, months 1–8 of pregnancy were defined as consecutive 4-week periods beginning on the estimated conception date; month 9 of pregnancy began on day 239 of gestation and ended on the day of birth (mean length of time, 40 days; range, 21–76 days).

In a complementary approach to the five-county population-weighted spatial average exposure assignment described above, we created spatial capture areas around each monitor and conducted monitor-specific analyses for the cohorts of infants with residential geocodes within 4 miles of each monitoring station. We limited these analyses to monitors that recorded daily pollutant concentrations.

### Analytic approach

Linear regression models were fitted to assess the relationship between continuous birth weight in full-term infants and air pollutant concentrations during the gestational windows of interest. We examined pollutants as linear terms in single-pollutant models. In the spatial capture area approach, separate linear regression models were fitted for each capture area, and effect estimates were pooled using inverse-variance weights to obtain a summary estimate and 95% confidence interval. All analyses were conducted using SAS version 9.2 (SAS Institute Inc., Cary, NC).

Because ambient air pollution levels exhibit strong seasonal variation, and other risk factors for reduced birth weight may also vary by season (Darrow et al. 2009b), we smoothly controlled for seasonal trends using parametric cubic splines on the date of conception. We adjusted for long-term temporal trends in birth weight using a second cubic spline with one knot per year; a cubic term without knots was used in the distributed lag analyses. We also assessed potential confounding by mean temperature and mean dew point over the gestational window of interest.

Although individual-level risk factors for reduced birth weight are unlikely to be confounders of the air pollution and birth weight relationship in this temporal analysis, individual-level predictors of birth weight were included to improve precision. Covariates included maternal education (< 12 years, 12–15 years, or ≥ 16 years of education), parity (first born vs. second or higher), race/ethnicity (Hispanic, non-Hispanic white, non-Hispanic black), marital status, reported tobacco use, infant sex, and maternal age (linear spline with knots at 20 and 35 years).

Finally, using interaction terms we examined effect modification of air pollution by race and ethnicity (Hispanic, non-Hispanic white, non-Hispanic black) to investigate the hypothesis that certain racial or ethnic groups might have differing susceptibility to ambient air pollution.

## Results

In the five-county cohort, the mean ± SD birth weight among full-term infants was 3,405 ± 477 g; for births within 4 miles of a monitor, the mean birth weight was 3,350 ± 475 g. Table 1 shows maternal and infant characteristics for these cohorts. Compared with the full five-county cohort, mothers residing within 4 miles of a monitor were younger, less educated, and more likely to be non-Hispanic black and unmarried.

Table 2 presents descriptive statistics of the population-weighted pollutant averages for the two gestational windows of *a priori* interest. The interquartile ranges (IQRs) of the pollutant concentrations differed slightly between the exposure windows for some pollutants; we used the IQRs presented in Table 2 to scale the effect estimates presented in later tables. Correlations between the pollutants for each gestational window are presented in Supplemental Material [Tables 1 and 2 (doi:10.1289/ehp.1002785)].

### Five-county analysis

Table 3 shows the change in birth weight per IQR increase in pollutant concentration during the first month of pregnancy and the third trimester. Results for the first month of pregnancy exposure window were consistent with little or no association; we observed one association between PM_2.5_ nitrate concentration and increased birth weight (*p* < 0.05). Third-trimester concentrations of NO_2_, SO_2_, PM_2.5_ elemental carbon, and PM_2.5_ water-soluble metals were associated with small decreases in birth weight ranging from −3.9 to −16.5 g per IQR increase in pollutant concentration. The association between third-trimester CO concentrations and birth weight (−7.1 g per IQR) was borderline significant (*p* = 0.06). When we examined third-trimester exposures for infants stratified by gestational week (37–44), pollutant effect estimates were similar between longer and shorter gestations. Mean temperature and dew point were not predictive of birth weight in either gestational window and did not meaningfully change the effect estimates for pollution, so they were not included in the final models.

**Figure 1:**
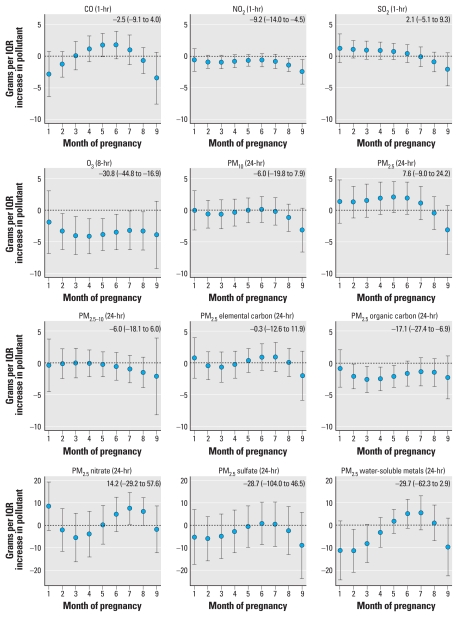
Associations between ambient pollutant concentrations and birth weight distributed across the months of pregnancy. The estimated change in birth weight and the 95% confidence interval displayed in the upper right-hand corner of each plot correspond to an IQR increase in the cumulative air pollution concentration during the entire pregnancy period. Estimates and 95% confidence intervals are presented graphically for the month-specific effects.

Figure 1 shows results from the constrained distributed lag models. Estimated month-specific effects reflect associations between pollutant concentrations and birth weight in each month of pregnancy, controlling for concentrations of the same pollutant in the other months of pregnancy. We scaled month-specific effect estimates to IQR increases in 4-week pollutant concentrations as shown in Table 2. Overall, results were suggestive of air pollutant concentrations in the ninth month of pregnancy being associated with reductions in birth weight, although only the association for NO_2_ was statistically significant. We used monthly estimates to estimate the cumulative effect of an IQR increase in pollutant concentrations across all months of pregnancy as shown in the upper right-hand corner of each graph in Figure 1. An IQR increase in NO_2_ concentrations during the 9-month pregnancy period (5 ppb) was associated with a 9.2-g decrease in birth weight; an IQR increase in O_3_ in all months of pregnancy (23 ppb) was associated with a 30.8-g decrease in birth weight; and an IQR increase in PM_2.5_ organic carbon in all months of pregnancy (1.2 μg/m^3^) was associated with a 17.1-g decrease in birth weight. Unconstrained distributed lag results (i.e., without forcing a cubic polynomial shape across months) were less smooth across months but overall showed similar patterns.

Table 4 shows the results from models assessing effect modification by race and ethnicity for pollutant concentrations in the third trimester. The pollution × race interaction terms were statistically significant for CO, O_3_, PM_10_, PM_2.5_, and PM_2.5–10_. Overall, we observed more statistically significant associations between air pollutants and birth weight in the Hispanic and non-Hispanic black groups than in the non-Hispanic white group. Notably, the association between PM_2.5_ water-soluble metals and birth weight was statistically significant and of similar magnitude for all three racial and ethnic groups.

### Capture-area analysis

Table 5 presents the change in birth weight and 95% confidence intervals per IQR increase in pollutant concentrations for the population of pregnancies within 4 miles of a monitor. We observed associations between third-trimester PM_2.5–10_ and increased birth weight and between CO concentrations in the first month of gestation and decreased birth weight. Although confidence intervals were wide and largely compatible with the estimates observed in the five-county analysis, these estimates provided little additional support for the associations observed in five-county population.

## Discussion

We investigated relationships between ambient concentrations of 12 pollutants during two *a priori* gestational windows of interest and birth weight in full-term infants. In the five-county analysis, we observed little evidence of an association between air pollution concentrations in the first month of gestation and reduced birth weight; we observed one association between PM_2.5_ nitrate and increased birth weight. Third-trimester concentrations of four pollutants (NO_2_, SO_2_, PM_2.5_ elemental carbon, and PM_2.5_ water-soluble metals) were associated with small decreases in birth weight. The capture-area analyses, which used measurements close to the maternal residence, provided little support for the associations observed in the five-county analysis, although confidence intervals were wide. The capture-area analyses did show an association between CO levels in the first month of gestation and decreased birth weight.

Given the number of statistical tests conducted in this analysis and the potential for type 1 errors, results should be interpreted in the context of previous research. Previous studies have reported associations between traffic-related pollutants and measures of fetal growth such as birth weight and small for gestational age (Aguilera et al. 2009; Bell et al. 2007; Gouveia et al. 2004; Liu et al. 2003; Wilhelm and Ritz 2003, 2005). Our observed associations between third-trimester NO_2_ and PM_2.5_ elemental carbon concentrations and reduced birth weight provide some additional evidence of this relationship. However, the specific pollutants and gestational windows of vulnerability have been inconsistent across studies (Slama et al. 2008). The International Collaboration on Air Pollution and Pregnancy Outcomes has recently emerged as an organized effort to streamline the methods used and potentially reconcile some of the apparent differences in results across studies (Woodruff et al. 2010).

The magnitude of birth weight reductions we observed was modest, ranging from a decrease of 3.9 g to 16.5 g per IQR increase in pollutant concentration. In comparison, maternal active smoking during pregnancy is thought to reduce birth weight by an average of 250 g (U.S. Department of Health and Human Services 2001), and maternal passive smoking is thought to reduce birth weight by an average of 30 g (U.S. Department of Health and Human Services 2006). Although the observed reductions have little clinical significance for an individual infant, on a population level a downward shift in the birth weight distribution would result in an increased number of infants at low birth weight (< 2,500 g). Although we scaled the pollutant estimates to an IQR increase in pollutant concentration, the range of pollutant concentrations extends well beyond the IQR. In addition, observed associations may have been biased toward the null by measurement error, particularly for spatially heterogeneous pollutants such as NO_2_, CO, and PM_2.5_ elemental carbon.

One possible explanation for seemingly contradictory results for PM and fetal growth across geographic locations is the difference in chemical composition and corresponding toxicity of PM across study sites (Parker and Woodruff 2008). Because of limited monitoring data, previous investigators have been unable to assess measures of reduced fetal growth in relation to specific PM_2.5_ components. A major strength of this study is the availability of daily measurements of PM_2.5_ component data between 1998 and 2004. PM_2.5_ elemental carbon and PM_2.5_ water-soluble metal concentrations in the third trimester were significantly associated with reduced birth weight, whereas other components of PM were not. In the distributed lag analyses, PM_2.5_ organic carbon concentrations throughout pregnancy were associated with decreased birth weight. The strongest association we observed was with third-trimester PM_2.5_ water-soluble metals, a sum of chromium, copper, iron, manganese, nickel, and vanadium. These metals have been shown to accumulate in fetal tissues and at high levels may have direct effects on fetal growth (Domingo 1994). Alternatively, our water-soluble metals index may be acting as a surrogate for other unmeasured pollutants from the same sources—for example, metal refining (chromium, iron, manganese), fuel oil combustion (nickel, vanadium), or motor vehicles (copper from brake linings).

Results of our analysis also suggested effect modification by race and ethnicity. Associations between birth weight and third-trimester CO, O_3_, PM_10_, PM_2.5–10_, and PM_2.5_ were significantly stronger in Hispanics and non-Hispanic African Americans than in non-Hispanic whites. In Connecticut and Massachusetts, associations between PM_2.5_ concentrations during pregnancy and birth weight were stronger in black women compared with white women (Bell et al. 2007). Another study in the northeastern United States also reported a stronger association between CO concentrations during pregnancy and low birth weight in African Americans (Maisonet et al. 2001). Differential susceptibility could be due to increased personal exposure to ambient air pollution in certain racial and ethnic groups (e.g., access to air conditioning) or differential biological sensitivity to air pollution stemming from differences in underlying health status, access to health care, or psychosocial stress (O’Neill et al. 2003). More research is needed to investigate potential differences in susceptibility among racial and ethnic population subgroups.

A major challenge in the field of air pollution and pregnancy outcomes has been identifying gestational windows of vulnerability. Because the correct gestational window(s) of exposure are unknown, we conducted distributed lag analyses, a data-based approach that may help to identify vulnerable gestational windows, particularly when exposures are correlated across the gestational windows. This approach has not previously been applied in the context of air pollution and pregnancy outcomes to our knowledge. Results of our analyses suggested that late pregnancy, specifically the ninth month, is a gestational window of vulnerability to air pollution. Evidence of early pregnancy as a vulnerable gestational window for air pollution effects on birth weight was weaker, with the exception of an association with first-month CO concentrations observed in the capture-area analysis. This could be a consequence of our inability to identify all conceptions, because we could identify only fetuses who survived to 20 weeks. If exposure to air pollution in early pregnancy increases the risk of fetal loss in addition to restricting fetal growth, associations between air pollution and birth weight would be underestimated. It is also possible that air pollution exposures influence fetal growth throughout gestation, but we were able to detect associations with birth weight only for exposures in the end of gestation because there is limited opportunity for catch-up growth between late exposure windows and birth. Studies that examine repeated measures of fetal growth throughout pregnancy rather than birth weight overcome this problem.

Measurement error in the ambient air pollution concentrations is a limitation of our study as well as previous studies in this field. In the temporal analytic setting, this error is attributable to both instrument imprecision and spatial variability, which are expected to attenuate associations (Goldman et al. 2009; Zeger et al. 2000). This might explain some of the null results we observed but would be unlikely to induce spurious associations. Concerns about exposure measurement error motivated our capture-area analyses, in which mothers were assigned pollutant concentrations measured within 4 miles of their residential address. However, with the exception of first-month CO, observed associations were not stronger in the capture-area analyses than in the five-county analysis, and point estimates provided little support for associations between local air pollutant concentrations and reduced birth weight. These findings do not strengthen conclusions regarding the harmful effect of air pollutant concentrations in Atlanta on birth weight suggested by the five-county analysis. However, it is possible that the local ambient monitors did not provide a better exposure measure for the capture-area population. Spatial variability of pollutant concentrations can be substantial even within 4 miles for vehicle emissions such as CO, NO_x_, and PM_2.5_ elemental carbon. Depending on the location of the monitor and the degree to which it is affected by local sources versus regional background, residential proximity to a monitor does not guarantee that air quality observations at that site are a better exposure measure. Pregnant women may also spend a large portion of their day away from their residence, and with approximately 20% of women in Atlanta changing residences during pregnancy, exposure assignment based on the residence at birth is problematic for assessment of early gestational windows (Miller et al. 2010).

In all of the analyses presented here, we made comparisons across time to reduce the plausibility of confounding by individual-level risk factors, which are unlikely to be associated with short-term fluctuations in air pollution (Strickland et al. 2009). This approach complements the existing literature, which is primarily based on spatiotemporal contrasts of exposure. Although we controlled for long-term temporal trends and seasonal trends and assessed confounding by meteorological variables, it is possible that results were confounded by other unmeasured risk factors that are associated with temporal fluctuations in air pollutant concentrations.

## Conclusion

We observed associations between air pollutant concentrations in late pregnancy and small reductions in birth weight in full-term infants. However, conclusions are somewhat tempered by the lack of support for these associations when air quality concentrations measured closer to the maternal residence were used. Our assessment of PM_2.5_ chemical components suggests that associations between PM and pregnancy outcomes may vary by chemical composition of the particles. Observed associations between birth weight and traffic-related pollutants (i.e., NO_2_ and PM_2.5_ elemental carbon) during the final trimester of pregnancy suggest that traffic sources of pollution should be further investigated in relation to fetal growth.

## Figures and Tables

**Table 1:** Maternal and infant characteristics [*n* (%)] for full-term births in five-county Atlanta and for births within 4 miles of a monitoring station, 1 January 1994 to 31 December 2004.

Characteristic	Five-county Atlanta (*n* = 406,627)	Births within 4 miles of a monitor (*n* = 110,357)
Term low birth weight (< 2,500 g)	10,615 (2.6)	3,601 (3.3)
Female sex	200,748 (49.4)	54,590 (49.5)
Maternal age group (years)		
< 20	42,420 (10.4)	15,282 (13.9)
20–34	303,755 (74.7)	80,113 (72.6)
≥ 35	60,452 (14.9)	14,962 (13.6)
Maternal race/ethnicity		
Non-Hispanic white	183,946 (45.2)	29,962 (27.2)
Non-Hispanic black	164,731 (40.5)	63,712 (57.7)
Hispanic	57,950 (14.3)	16,683 (15.1)
Maternal education (completed years)		
< 12	78,926 (19.4)	28,761 (26.1)
12–15	189,818 (46.7)	50,709 (45.9)
≥ 16	137,883 (33.9)	30,887 (28.0)
Married	262,769 (64.6)	55,574 (50.4)
First birth	176,592 (43.4)	48,763 (44.2)
Reported tobacco use during pregnancy	19,236 (4.7)	4,603 (4.2)

**Table 2:** Descriptive statistics of the assigned pollutant values in the five-county cohort.

	First month of gestation (4-week average)		Third trimester	
Pollutant	Mean ± SD	IQR	Mean ± SD	IQR
1-hr maximum concentration^a^				
CO (ppm)	0.9 ± 0.2	0.3	0.8 ± 0.2	0.3
NO_2_ (ppb)	23.6 ± 4.0	5	23.8 ± 3.3	5
SO_2_ (ppb)	10.7 ± 3.4	4	9.5 ± 2.0	3
8-hr maximum O_3_ (ppb)^a^	44.8 ± 15.3	25	44.2 ± 12.5	23
24-hr PM concentration (μg/m^3^)				
PM_10_^a^	23.8 ± 6.3	8	23.4 ± 4.2	7
PM_2.5_^a^	16.5 ± 4.1	5	16.4 ± 3.1	4
PM_2.5–10_^b^	9.1 ± 2.5	2.7	9.0 ± 1.7	2.0
PM_2.5_ sulfate^b^	4.9 ± 2.3	2.9	5.1 ± 1.8	2.9
PM_2.5_ nitrate^b^	1.0 ± 0.5	0.7	0.9 ± 0.4	0.6
PM_2.5_ elemental carbon^b^	1.6 ± 0.5	0.5	1.6 ± 0.4	0.5
PM_2.5_ organic carbon^b^	4.4 ± 1.0	1.2	4.3 ± 0.6	1.0
PM_2.5_ water-soluble metals^b^,^c^	0.030 ± 0.013	0.017	0.031 ± 0.010	0.016

Pollutant monitoring methods: CO, infrared analyzer; NO_2_, chemiluminescence; SO_2_, fluorescence; O_3_, ultraviolet absorption; PM_10_, federal reference method (eight sites) and tapered element oscillating microbalance (one site); PM_2.5_, federal reference method (eight sites) (U.S. EPA 1997) and tapered element oscillating microbalance (three sites); PM_2.5–10_, dichotomous sampler; PM_2.5_ components, particle composition monitor; sulfate and nitrate, ion chromatography; elemental and organic carbon, thermal optical reflectance; and water-soluble metals, x-ray fluorescence. For more details on air quality measurements and instrumentation see Hansen et al. (2006) and Ivy et al. (2008).

a Concentrations reflect population-weighted ambient pollution values.

b Concentrations reflect central monitor measurements.

c Water-soluble metal index includes chromium, copper, iron, manganese, nickel, and vanadium.

**Table 3:** Mean change in birth weight (Δg) and 95% confidence intervals (CI) for ambient air pollutant concentrations in the first month of pregnancy and the third trimester for full-term births in five-county Atlanta.

	First month of gestation		Third trimester (week 28 to birth)	
Pollutant	*n*^a^	Δg (95% CI)	*n*^a^	Δg (95% CI)
1-hr maximum concentration				
CO (ppm)	406,616	−1.8 (−5.7 to 2.2)	406,627	−7.1 (−14.5 to 0.2)
NO_2_ (ppb)	404,195	0.4 (−1.8 to 2.6)	406,627	−4.5 (−8.5 to −0.6)*
SO_2_ (ppb)	400,556	0.5 (−2.1 to 3.1)	406,627	−3.9 (−7.5 to −0.4)*
8-hr maximum O_3_ (ppb)	406,627	−1.2 (−7.5 to 5.2)	406,627	−2.2 (−11.2 to 6.9)
24-hr PM concentration (μg/m^3^)				
PM_10_	311,365	1.0 (−2.1 to 4.0)	333,190	−2.3 (−7.4 to 2.8)
PM_2.5_	225,522	0.0 (−3.7 to 3.7)	243,481	−4.3 (−9.8 to 1.2)
PM_2.5–10_	194,683	−2.2 (−5.7 to 1.4)	215,136	−3.1 (−7.6 to 1.4)
PM_2.5_ sulfate	182,290	0.8 (−4.5 to 6.1)	194,677	−8.5 (−19.1 to 2.0)
PM_2.5_ nitrate	181,831	9.2 (3.0 to 15.4)*	185,597	−5.5 (−15.4 to 4.5)
PM_2.5_ elemental carbon	220,867	0.7 (−2.4 to 3.8)	243,195	−7.1 (−13.9 to −0.3)*
PM_2.5_ organic carbon	220,867	−0.1 (−3.1 to 2.9)	243,195	−3.4 (−8.5 to 1.7)
PM_2.5_ water-soluble metals	183,316	2.5 (−3.5 to 8.5)	193,377	−16.5 (−28.4 to −4.7)*

Change in birth weight and 95% CIs correspond to an IQR increase in pollutant value shown in Table 2. Models controlled for long-term trends, seasonal trends, gestational week, sex, and maternal marital status, education, age, race/ethnicity, reported tobacco use, and parity.

a Number of infants with an assigned exposure.

* *p* < 0.05.

**Table 4:** Mean change in birth weight (Δg) and 95% confidence intervals (CI) for ambient air pollution levels in the third trimester for full-term births in five-county Atlanta by race and ethnicity.

	Non-Hispanic white		Non-Hispanic black		Hispanic	
Pollutant	*n*	Δg (95% CI)	*n*	Δg (95% CI)	*n*	Δg (95% CI)
1-hr maximum concentration						
CO (ppm)*	183,946	−5.6 (−13.3 to 2.1)	164,731	−6.6 (−14.3 to 1.2)	57,950	−14.4 (−23.4 to −5.5)**
NO_2_ (ppb)	183,946	−4.6 (−9.3 to 0.1)	164,731	−3.9 (−8.7 to 0.8)	57,950	−5.8 (−12.4 to 0.7)
SO_2_ (ppb)	183,946	−5.2 (−9.2 to −1.2)**	164,731	−1.9 (−5.9 to 2.2)	57,950	−5.7 (−11.4 to −0.1)**
8-hr maximum O_3_ (ppb)*	183,946	4.3 (−5.1 to 13.6)	164,731	−7.6 (−17.1 to 1.8)	57,950	−11.1 (−22.1 to −0.0)**
24-hr PM concentration (μg/m^3^)						
PM_10_*	144,761	3.5 (−2.2 to 9.1)	135,077	−5.5 (−11.2 to 0.2)	53,352	−12.1 (−19.6,−4.7)**
PM_2.5_*	100,562	0.1 (−6.0 to 6.2)	97,861	−6.3 (−12.5 to −0.2)**	45,058	−10.2 (−17.6 to −2.7)**
PM_2.5–10_*	88,135	2.3 (−2.9 to 7.5)	86,225	−5.4 (−10.6 to −0.2)**	40,776	−8.6 (−14.8 to −2.5)**
PM_2.5_ sulfate	79,275	−5.2 (−16.3 to 5.9)	77,894	−11.7 (−22.9 to −0.6)**	37,508	−9.4 (−22.0 to 3.2)
PM_2.5_ nitrate	75,193	−10.3 (−21.0 to 0.3)	74,170	−2.7 (−13.3 to 7.9)	36,234	−2.1 (−13.8 to 9.4)
PM_2.5_ elemental carbon	100,446	−5.5 (−12.8 to 1.7)	97,727	−7.9 (−15.2 to −0.7)**	45,022	−8.6 (−17.1 to −0.2)**
PM_2.5_ organic carbon	100,446	−3.4 (−9.5 to 2.8)	97,727	−3.2 (−9.3 to 3.0)	45,022	−3.9 (−11.8 to 3.9)
PM_2.5_ water-soluble metals	78,715	−14.7 (−27.1 to −2.3)**	77,418	−17.4 (−29.8 to −5.0)**	37,244	−18.7 (−32.2 to −5.1)**

Change in birth weight and 95% CIs correspond to an IQR increase in third-trimester pollutant concentration reported in Table 2. Models controlled for long-term trends, seasonal trends, gestational week, sex, and maternal marital status, education, age, race/ethnicity, reported tobacco use, and parity.

* *p* < 0.05 for chunk test of interaction terms.

** *p* < 0.05 for pollutant beta.

**Table 5:** Mean change in birth weight (Δg) and 95% confidence intervals (CI) for ambient air pollution levels in selected gestational windows for births with a maternal residential address within 4 miles of a monitor.

Pollutant	First month of gestation		Third trimester		*n* monitor capture areas^b^
	*n*^a^	Δg (95% CI)	*n*^a^	Δg (95% CI)	
1-hr maximum concentration					
CO (ppm)	45,985	−5.2 (−10.0 to −0.5)*	44,226	1.8 (−6.5 to 10.3)	3
NO_2_ (ppb)	48,112	−1.6 (−5.7 to 2.5)	46,251	3.6 (−3.7 to 11.0)	4
8-hr maximum O_3_ (ppb)	29,281	6.8 (−13.3 to 26.9)	26,054	5.8 (−27.1 to 38.7)	3
24-hr PM concentration (μg/m^3^)					
PM_10_	16,512	2.2 (−11.3 to 15.6)	16,384	15.9 (−8.6 to 40.5)	2
PM_2.5_	37,354	−4.2 (−13.6 to 5.2)	35,026	−2.5 (−20.4 to 15.3)	6
PM_2.5–10_	11,259	−0.6 (−15.0 to 13.9)	12,570	21.0 (2.6 to 39.4)*	1
PM_2.5_ sulfate	10,518	−1.8 (−23.5 to 20.0)	11,360	−11.8 (−54.4 to 30.9)	1
PM_2.5_ nitrate	10,515	−5.2 (−30.8 to 20.5)	10,822	−3.0 (−44.2 to 38.2)	1
PM_2.5_ elemental carbon	12,918	−2.3 (−14.7 to 10.2)	14,292	4.6 (−23.2 to 32.4)	1
PM_2.5_ organic carbon	12,918	−4.4 (−16.6 to 7.9)	14,292	2.7 (−18.2 to 23.5)	1
PM_2.5_ water-soluble metals	10,576	14.5 (−9.7 to 38.6)	11,290	−8.4 (−55.5 to 38.8)	1

Change in birth weight and 95% CIs correspond to an IQR increase in pollutant value for each exposure window reported in Table 2. Models control for long-term trends, seasonal trends, gestational week, sex, and maternal marital status, education, age, race/ethnicity, reported tobacco use, and parity.

a Number of births in pooled analysis.

b Only monitors with daily measurements were used.

* *p* < 0.05.
